# Supplementary material for: Deletion of wfs1 Impairs Oligodendrocyte Precursor Cells Dorsal Distribution and Myelination Through the wfs1-hmgcs1 Axis in Zebrafish
Source: Biology (Basel). 2026 Aug 16;15(16):1402. doi: 10.3390/biology15161402 (PMC13510151; doi:10.3390/biology15161402)
Supplement: Supplementary file 1 [file biology-15-01402-s001.zip › File S1-Transcriptome sequencing data and differentially expressed genes (DEGs) in wfs1-- zebrafish at 5 dpf/2.KEGG/wfs1KOvsWT/UP/src/dre04140.html]

dre04140


- dre:245702

- Up regulated genes

245702(1.27755220735994)

- dre:100000252

- Up regulated genes

100000252\_3(1.94434154854557) 100000252(1.09163460881879)

- dre:100330617

- Up regulated genes

100330617\_1(1.54741913113035)

- dre:406843

- Up regulated genes

406843\_1(1.98494944295698)

- dre:573492

- Up regulated genes

573492\_1(2.04323918457063)

- dre:567021

- Up regulated genes

567021(7.12614903992843)

- dre:792229

- Up regulated genes

792229\_1(2.08412818777288)

- dre:563140

- Up regulated genes

563140(1.55773055978699)

- dre:110438871

- Up regulated genes

110438871(1.82412391238383)

- dre:326974

- Up regulated genes

326974\_1(1.07837684837292)

- dre:449923

- Up regulated genes

449923(3.08455507116647) 449923\_1(1.39798369726066)

- dre:326974

- Up regulated genes

326974\_1(1.07837684837292)

- dre:569298

- Up regulated genes

569298(1.32871616109562)

Close
